# Metabolic Profile Reveals the Immunosuppressive Mechanisms of Methionyl-Methionine in Lipopolysaccharide-Induced Inflammation in Bovine Mammary Epithelial Cell

**DOI:** 10.3390/ani11030833

**Published:** 2021-03-16

**Authors:** Wei Lan, Yifei Ren, Zhen Wang, Jianxin Liu, Hongyun Liu

**Affiliations:** College of Animal Sciences, Zhejiang University, Hangzhou 310058, China; wei-lan@zju.edu.cn (W.L.); 21717016@zju.edu.cn (Y.R.); 21817007@zju.edu.cn (Z.W.); liujx@zju.edu.cn (J.L.)

**Keywords:** Met-Met, lipopolysaccharide, inflammation, MAC-T

## Abstract

**Simple Summary:**

Bovine mastitis results in substantial problems in terms of animal health, food safety, and profitability of farmers. Methionyl-methionine dipeptide confers protection in the bovine mammary epithelial cells as evidenced by our previous transcriptomic study at the molecular level. However, whether the metabolic production of Met-Met confers protection remains unknown. In this study, Met-Met significantly suppressed LPS-induced TNF-α, IL-8, AP-1, and MCP-1 expression; reversed decreased tryptophan, phenylalanine, and histidine levels; and inhibited LPS-induced palmitic acid and stearic acid levels as well as purine metabolism disorder. Overlapping metabolites were mainly involved in the cysteine and methionine metabolism, fatty acids biosynthesis, and purines degradation. These metabolites and pathways might contribute to the protective action of methionyl-methionine.

**Abstract:**

Our previous transcriptomic study found that methionyl-methionine (Met-Met) exerts an anti-inflammatory effect in the bovine mammary epithelial cell (MAC-T) at a molecular level. However, evidence of whether the metabolic production of Met-Met confers protection was scarce. To investigate the inflammatory response and metabolite changes of Met-Met in lipopolysaccharide (LPS)-induced inflammation of MAC-T, mass spectrometry-based metabolomics and qPCR were conducted. The increased levels of *IL-8*, *TNF-α*, *AP-1,* and *MCP-1* were reduced by pretreating with 2 mM Met-Met after LPS exposure. Metabolomics profiling analysis demonstrated that LPS induced significant alteration of metabolites, including decreased tryptophan, phenylalanine, and histidine levels and increased palmitic acid and stearic acid levels as well as purine metabolism disorder, whereas Met-Met reversed these changes significantly. Pathways analysis revealed that overlapping metabolites were mainly enriched in the cysteine and methionine metabolism, fatty acids biosynthesis, and purines degradation. Correlation networks showed that the metabolic profile was significantly altered under the conditions of inflammation and Met-Met treatment. Collectively, Met-Met might relieve MAC-T cell inflammation via hydrolysate methionine, which further changes the processes of amino acid, purine, and fatty acid metabolism.

## 1. Introduction

With the intensive development of the dairy industry, mastitis caused by Gram-negative bacteria in the environment has attracted much attention [1]. For this reason, the pathogenic mechanism of Gram-negative bacteria in dairy cows is fundamental to control bovine mastitis. Previous studies have shown that lipopolysaccharide (LPS), as the main component of the cell wall of Gram-negative bacteria, is an important part of inducing mastitis in dairy cows [2]. Lipopolysaccharide can bind to toll-like acceptor 4 (TRL4) receptors on the cell membrane of bovine mammary epithelial cells (MAC-T), and then further activate the nuclear factor kappa-B (NF-κB) and mitogen-activated protein kinase (MAPK) signaling pathways to induce mastitis [3,4].

Several efficient programs are utilized to control bovine mastitis. At present, most of the clinical treatments for mastitis resulting from Gram-positive bacteria (such as *Streptococci* and *Staphylococci*) are based on targeted antibiotics [5]. However, intramammary antibiotic treatment of clinical mastitis (CM) caused by *Escherichia coli* (*E. coli*) is not satisfactory, becase the use of extended-duration therapy to treat *E. coli* significantly increases costs without improving clinical outcomes of mastitis [6,7,8]. Therefore, despite the fulfillment of control programs, the commonness of bovine mastitis is a major problem. Notably, a study focusing on improving cow immunity by nutritional additives, such as dipeptides, instead of drug treatment is superior.

Methionyl-methionine dipeptide (Met-Met) contains two DL-methionines that modulate bovine blood polymorphonuclear cells’ inflammatory status and help the resolution of inflammation after LPS stimulation [9]. Besides, compared with the uptake of free methionine, the uptake of Met-Met by dipeptide transporters located on the cell membrane is more efficient [10,11], as evidenced by a study that Met-Met could provide two free methionine for the synthesis of milk protein in mammary gland tissue explants [12]. Furthermore, Met-Met can be absorbed by cells independent of free methionine and is more stable in water than free methionine, thus it is widely used in aquatic animals [13]. For example, compared with free methionine, Met-Met is better absorbed to improve fish intestinal immune function [14]. Our previous transcriptomic study found that Met-Met exhibited an anti-inflammatory effect via activating the genes and proteins related to inflammation in MAC-T cells [15]. However, whether the metabolic production of Met-Met confers protection remains obscure. Thus, the purpose of this study was to determine the inflammatory response and metabolic changes in MAC-T cells through a metabolome analysis to further unravel the beneficial effect of Met-Met.

## 2. Materials and Methods 

### 2.1. Reagents

Met-Met was provided by ChinaPeptides Co., Ltd. (Suzhou, China). The certificates of mass spectrometry (MS) and high-performance liquid chromatography (HPLC) analysis of Met-Met are uploaded to the Supplemental Materials. LPS (*E. coli* O111:B4) was purchased from Sigma Company (St. Louis, MO, USA).

### 2.2. Cell Study

The MAC-T was cultured in Dulbecco’s modified Eagle’s medium (Gibco, NY, USA) containing 1% penicillin/streptomycin (Keyi Biotech, Hangzhou, China), 5 μg/mL insulin (Sigma), 1 μg/mL hydrocortisone, and 10% fetal bovine serum (FBS) (Gibco) [16]. The final content of 2 mM Met-Met and 1 μg/mL LPS in culture medium and experimental treatments were chosen based on our previous studies [15]. As for the experiment of the inflammatory response, MAC-T was seed into a 6-well plate at the density of 1 × 10^6^ cells/mL and incubated with or without Met-Met (2 mM) for 6 h and then stimulated by fresh media containing LPS (1 μg/mL) for 1, 3, 6, and 12 h. For the metabolic experiment, MAC-T was treated with Met-Met (2 mM) for 6 h and further co-incubated with 1 μg/mL LPS for 3 h, and then the supernatant was removed and the cells were collected for metabolomic analysis. Each experiment was divided into four groups, i.e., the control group (CON, -Met-Met-LPS), inflammation group (LPS, -Met-Met+LPS), anti-inflammatory group (Met-Met+LPS, +Met-Met+LPS), and dipeptide group (Met-Met, +Met-Met-LPS). Finally, MAC-T was obtained for further analyses.

### 2.3. Real-Time PCR (RT-PCR)

The total RNA of MAC-T was collected using Trizol reagent (Invitrogen, Carlsbad, CA, USA). The quality of total RNA was assessed by an Agilent 2100 (Agilent Technologies, Santa Clara, USA), and the samples with an RNA integrity number (RIN) larger than 7.6 were selected for subsequent experiments. The total RNA (1 μg) was reverse transcribed into cDNA by the PrimeScript RT reagent kit (TaKaRa, Dalian, China). Quantitative RT-PCR was carried out in the 7500 real-time PCR system (Applied Biosystems, Foster City, CA) with SYBR Premix Ex Taq (TaKaRa). PCR conditions were as follows: 95 °C for 30 s, 40 cycles of 95 °C for 5 s, and 60 °C for 34 s. The specificity of amplification was evaluated by the melt curve of an additional 15 s at 95 °C, 1 min at 60 °C, and 15 s at 95 °C. Several genes related to cytokines (tumor necrosis factor-α (*TNF-α*), interleukin 8 (*IL-8*)) and inflammatory mediators (activator protein 1 (*AP-1*), monocyte chemotactic protein 1 (*MCP-1*)) were selected to evaluate the inflammatory response after LPS stimulation. The relative expression of genes was normalized to the special internal reference ribosomal protein S9 (RPS9) [17,18,19]. The mRNA levels of the genes were calculated according to the 2^−∆∆CT^ method. The primer sequences of the genes are described in Table S1. Among them, the primer sequences of *TNF-α, IL-8,* and *RPS9* refer to previous studies [20,21].

### 2.4. Sample Preparation for Liquid Chromatography-Mass Spectrometry (LC/MS) Detection

In all, 12 samples (4 groups × 3 samples per group) were delivered to Shanghai Applied Protein Technology Co., Ltd. (Shanghai, China) for liquid chromatography-tandem mass spectrometry (LC-MS/MS) analysis. Every sample was thawed at 4 °C, mixed with 1 mL of cold methanol/acetonitrile/H_2_O (2:2:1, *v*/*v*/*v*), and adequately vortexed. The homogenate was sonicated at 4 °C (30 min/once × 2), incubated for 60 min at −20 °C to precipitate the protein, and centrifuged (13,000 rpm, 4 °C, 15 min). The supernatants were collected and dried under vacuum and then stored at −80 °C. The sample was redissolved in 100 μL of acetonitrile/water (1:1, *v*/*v*), adequately vortexed, and then centrifuged (14,000 rpm, 4 °C, 15 min). The supernatants were collected for LC-MS/MS analysis.

### 2.5. LC/MS-Based Cell Metabolic Profiling

All samples were analyzed using a chromatographic column (ACQUIY UPLC BEH (100 mm × 2.1 mm 1.7 µm)). Column temperature (25 °C) and flow rate (0.3 mL/min) were selected as separation conditions. Mobile phase A was 25 mM ammonium acetate and 25 mM ammonium hydroxide in water (CNW, Duesseldorf, Germany), while mobile phase B was acetonitrile (Merck, Darmstadt, Germany). The gradient was 85% B for 1 min and was linearly reduced to 65% in 11 min, then reduced to 40% in 0.1 min and kept for 4 min, and then increased to 85% in 0.1 min, with a 5-min re-equilibration period employed. After separation by ultra-high performance liquid chromatography (UHPLC), all samples were analyzed by a Triple TOF 6600 mass spectrometer (AB SCIEX, Danaher, Washington, DC, USA). The parameters of the MS were as follows: ion source gas 1, 60; ion source gas 2, 60; curtain gas, 30; source temperature, 600 °C; and ion spray voltage floating (ISVF), ±5500 V. In MS acquisition, the instrument was set to acquire over the m/z range 60–1000 Da, and the accumulation time was set at 0.20 s/spectra.

### 2.6. Data Processing and Differential Metabolites Screening

The metabolites were identified by comparing the MS/MS spectra with the spectra in the Kyoto Encyclopedia of Genes and Genomes database (https://www.genome.jp/kegg/, accessed on 20 July 2020) and Human Metabolome Database (http://www.hmdb.ca, accessed on 25 July 2020). The metabolites with high confidence were validated by comparing the retention time and MS/MS spectra of commercial reference standards. The LC-MS data were analyzed using SIEVE software (Thermo, Waltham, MA, USA). Partial least squares discriminant analysis (PLS-DA) and orthogonal partial least squares discriminant analysis (OPLS-DA) were performed using SIMCA-P 14.1 software (Umetrics, Umea, Sweden). Differential metabolites were detected according to the variable importance in the projection (VIP > 1.0) of PLS-DA and *p* values (*p* < 0.1) calculated by student’s t-test. Then, the differential metabolite data were uploaded into the MetaboAnalyst web server (http://www.metaboanalyst.ca, accessed on 25 July 2020) to determine the metabolic pathway distribution and to perform metabolite–metabolite interaction analysis.

### 2.7. Statistical Analysis

Significance differences in cytokine expression at the same time points were assessed using unpaired Student’s t-test in SPSS 20.0 (Chicago, IL, USA). * *p* < 0.05, ** *p* < 0.01, when comparing the LPS group with the CON group; ^#^ *p* < 0.05 and ^##^ *p* < 0.01 denote the significance between the Met-Met+LPS and LPS groups; and ^$^ *p* < 0.05, ^$$^ *p* < 0.01, when comparing the Met-Met group with the LPS group.

## 3. Results 

### 3.1. Effect of Met-Met on Inflammatory Response in MAC-T

**Figure 1:**
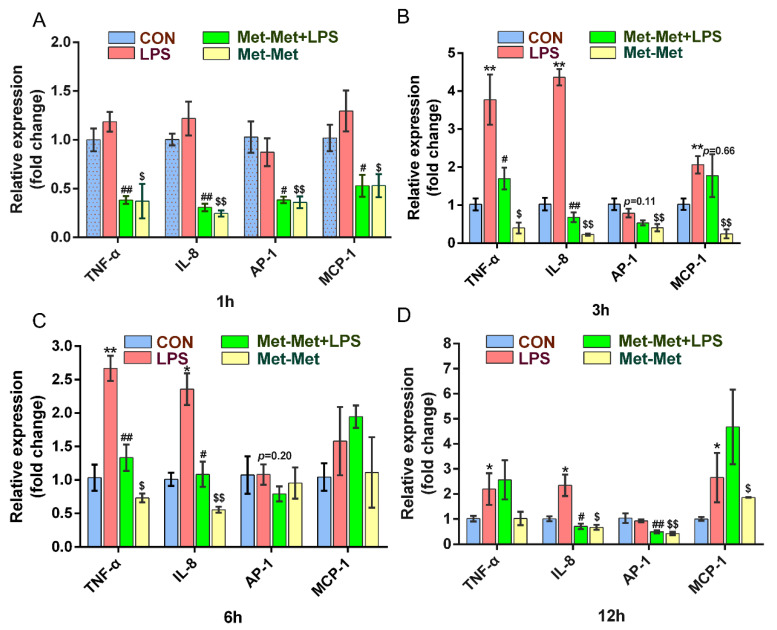
Effect of Met-Met on the inflammatory response in MAC-T. (**A**). MAC-T was incubated with or without Met-Met (2 mM) for 6 h and then stimulated by LPS (1 μg/mL) for 1 h to examine the mRNA expression of *TNF-α*, *IL-8*, *AP-1*, and *MCP-1*. (**B**). The mRNA expression of *TNF-α*, *IL-8*, *AP-1*, and *MCP-1* at 3 hours. (**C**). The levels of *TNF-α*, *IL-8*, *AP-1*, and *MCP-1* at 6 hours. (**D**). The abundance of *TNF-α*, *IL-8*, *AP-1*, and *MCP-1* at 12 hours. The means ± SEM (n = 3) were shown and calculated by unpaired Student’s t-test at the same time points. * *p* < 0.05 and ** *p* < 0.01 represent significance in the LPS vs. CON group; ^#^ *p* < 0.05 and ^##^ *p* < 0.01 denote the significance between the Met-Met+LPS and the LPS groups; ^$^ *p* < 0.05, ^$$^ *p* < 0.01, when compared the Met-Met group with the CON group. Tumor necrosis factor-α, *TNF-α; I*nterleukin 8, *IL-8*; Activator protein 1, *AP-1*; Monocyte chemotactic protein 1, *MCP-1.*

The levels of the cytokines *TNF-α* and *IL-8* were prominently higher at 3 (3.77 and 4.36-fold) and 6 h (2.65 and 2.36-fold) than those at 1 (1.18 and 1.21-fold) and 12 h (2.19 and 2.34-fold) when compared the LPS group with the CON group. Treatment with Met-Met +LPS significantly suppressed the expression of *TNF-α* at 1 (0.38 ± 0.07 vs. 1.18 ± 0.18, *p* < 0.01) (Figure 1A), 3 (1.69 ± 0.49 vs. 3.77 ± 1.14, *p* < 0.05) (Figure 1B), and 6 h (1.33 ± 0.34 vs. 2.67 ± 0.32, *p* < 0.01) (Figure 1C) except for the 12-h time points, when compared with the LPS group alone. Consistently, the levels of *IL-8* in the Met-Met+LPS group were strongly decreased at 1 (0.31 ± 0.07 vs. 1.22 ± 0.29, *p* < 0.01) (Figure 1A), 3 (0.68 ± 0.22 vs. 4.36 ± 0.37, *p* < 0.01) (Figure 1B), 6 (1.08 ± 0.32 vs. 2.36 ± 0.41, *p* < 0.05)(Figure 1C), and 12 h (0.70 ± 0.19 vs. 2.34 ± 0.74, *p* < 0.05) (Figure 1D) when compared with the LPS group. The expression of *AP-1* was significantly lower in the Met-Met+LPS group than the LPS goup at 1 (0.38 ± 0.05 vs. 0.87 ± 0.24, *p* < 0.05) (Figure 1A) and 12 h (0.49 ± 0.08 vs. 0.93 ± 0.10, *p* < 0.01) (Figure 1D). The level of *MCP-1* was suppressed at 1 h (0.53 ± 0.19 vs. 1.29 ± 0.36, *p* < 0.05) (Figure 1A) in the Met-Met+LPS group when compared with the LPS group; however, this suppression of MCP-1 by Met-Met decreased over time (Figure 1B–D). Interestingly, compared with the control group, Met-Met treatment alone significantly suppressed the expression of *TNF-α*, *IL-8*, *AP-1*, and *MCP-1* at 1 and 3 h (Figure 1A,B).

### 3.2. The MAC-T Cell Metabolomics Profile Is Affected by Met-Met

**Figure 2:**
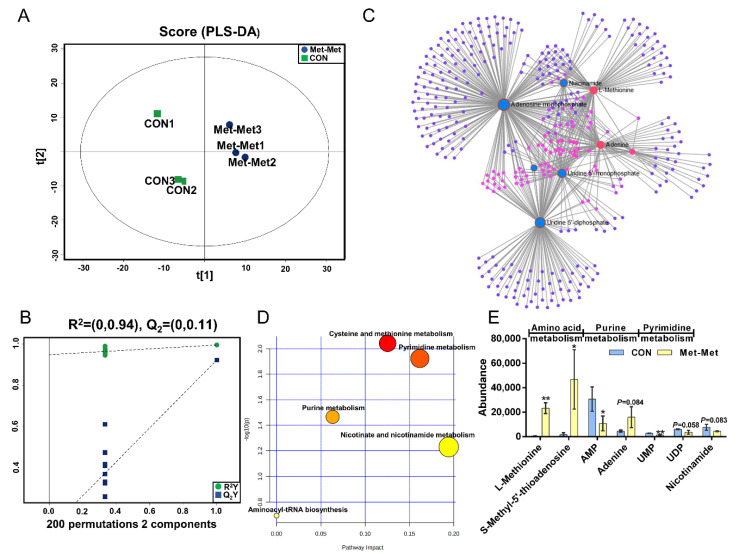
The MAC-T cell metabolomics profile is affected by Met-Met. (**A**). The PLS-DA score plot of the Met-Met vs. CON group. (**B**). Cross-validation plot of the PLS-DA model with 200 permutation tests in the Met-Met vs. CON group. (**C**). Metabolite-metabolite interaction network of DMs in the Met-Met vs. CON group. Node size represents the degree. Red and blue circles correspond to the increased and reduced DMs in the Met-Met vs. CON group, respectively. (**D**). Pathway analysis of DMs in the Met-Met vs. CON group. The *X*-axis represents the pathway impact, and the *Y*-axis represents the pathway enrichment degree. A darker color indicates a higher degree of pathway enrichment, and a larger size indicates a higher pathway impact value. (**E**). The classification map of DMs. * *p* < 0.05, ** *p* < 0.01, when compared the Met-Met with the CON group; DMs: differential metabolites.

PLS-DA (Figure 2A) and OPLS-DA (Figure S1A) of MAC-T in the Met-Met and CON groups showed that the cell metabolites of Met-Met were distinctly separated from those of the CON group. Generally, R^2^Y of PLS-DA provides an estimate of how well the model fits Y, and Q_2_ is an estimate of how well the model predicts X. Two hundred permutation test models (Figure 2B) showed good fitness and prediction with an R^2^Y value of 0.94 and a Q_2_ value of 0.11 for the cell sample data. Combined with variable importance in projection (VIP) and P-value (VIP >1 and *p* < 0.1), eight differential metabolites (DMs) were identified in Met-Met vs. CON, of which four were upregulated and four were downregulated (Table S2). The most significant DMs were L-methionine (57-fold), S-methyl-5’-thioadenosine (27-fold), and phenylacetic acid (10-fold). Details of the DMs, including name, VIP, P-value, fold change (FC), mass-to-charge ratio (M/Z), and retention time (Rt), are presented in Table S2. Correlation networks exhibit the comprehensive relationships between cell metabolites and overall metabolic profiles in a comparative group of Met-Met vs. CON. Nodes and edges represent metabolites and correlations in the correlation networks, respectively. Our correlation network (Figure 2C) consists of 370 nodes and 639 edges, revealing that Met-Met caused a large alteration of metabolites. The most correlated DMs are L-methionine, S-methyl-5’-thioadenosine, phenylacetic acid, adenine, adenosine monophosphate (AMP), and uridine-5’-monophosphate (UMP). Enrichment analysis was performed to comprehensively evaluate how multiple pathways were disturbed when the cell was treated with Met-Met. The KEGG analysis showed that a majority of metabolites were primarily involved in cysteine and methionine metabolism, pyrimidine metabolism, purine metabolism, nicotinate and nicotinamide metabolism, and aminoacyl-tRNA metabolism (Figure 2D). According to the biochemical characteristics and biological processes, these DMs and metabolic pathways could be further divided into three categories: amino acid metabolism, purine metabolism, and fatty acid metabolism (Figure 2E).

### 3.3. Metabolomics Analysis of Inflammation Response in MAC-T

**Figure 3:**
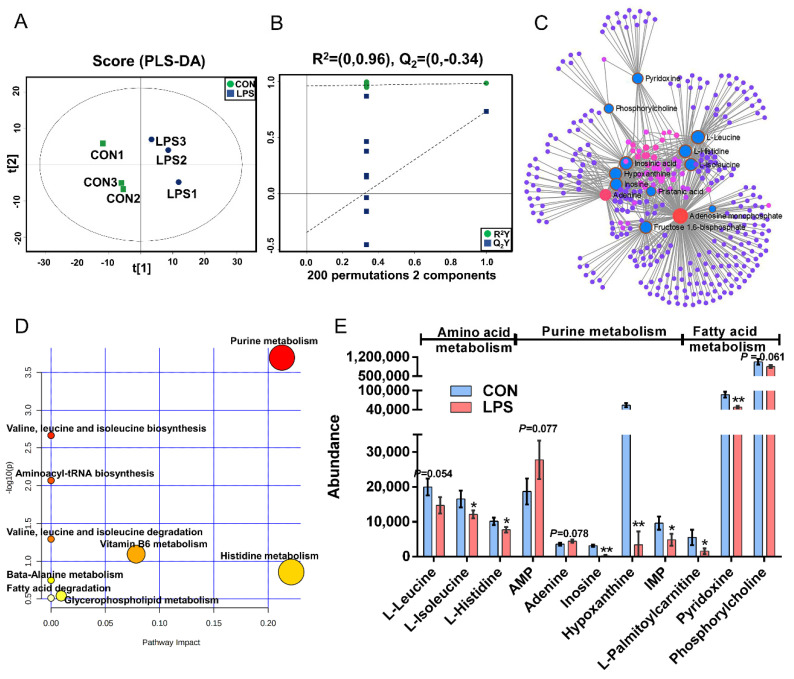
Metabolomics analysis of inflammation response in MAC-T. (**A**). The PLS-DA score plot of the LPS vs. CON group. (**B**). Cross-validation plot of the PLS-DA model with 200 permutation tests in the comparative group of LPS vs. CON; (**C**). Metabolite-metabolite interaction network of DMs in the comparative group of LPS vs. CON. Node size represents the degree. Red and blue circles correspond to the increased and reduced DMs in the LPS vs. CON group, respectively. (**D**). Pathway analysis of DMs in the LPS and CON groups. The *X*-axis and *Y*-axis indicate the pathway impact and the degree of pathway enrichment, respectively. A darker color indicates a higher degree of pathway enrichment, and a larger size indicates a higher pathway impact value. (**E**). The classification map of DMs and pathways. * *p* < 0.05 and ** *p* < 0.01, represent the difference between LPS and CON group; DMs: differential metabolites.

The PLS-DA (Figure 3A), OPLS-DA (Figure S1C), and 200 permutation test models (Figure 3B) of MAC-T in the LPS and CON groups showed that the cell metabolites of the inflammatory group were separated from those of the CON group. Combined with VIP >1 and *p* < 0.1, 14 DMs were identified in the LPS vs. CON group, of which 12 were downregulated and 2 were upregulated (Table S3). The most significant DMs were AMP (1.48-fold). Details of the DMs, including name, VIP, P-value, FC, M/Z, and Rt, are presented in Table S3. The correlation networks of the LPS vs. CON group (Figure 3C) consisted of 338 nodes and 729 edges. The most correlated DMs are AMP, adenine, leucine, histidine, isoleucine, phosphorylcholine, D-fructose-1,6-bisphosphate, inosinic acid, hypoxanthine, inosine, and palmitic acid. KEGG pathway analysis (Figure 3D) showed that purine metabolism, histidine metabolism, vitamin B6 metabolism, glycerophospholipid metabolism, and fatty acid degradation were significantly enriched (*p* < 0.05). According to the biochemical characteristics and biological processes, these DMs and metabolic pathways could be further divided into three categories: amino acid metabolism, purine metabolism, and fatty acid metabolism (Figure 3E).

### 3.4. Met-Met Altered the Metabolic Status of Inflammatory Response

**Figure 4:**
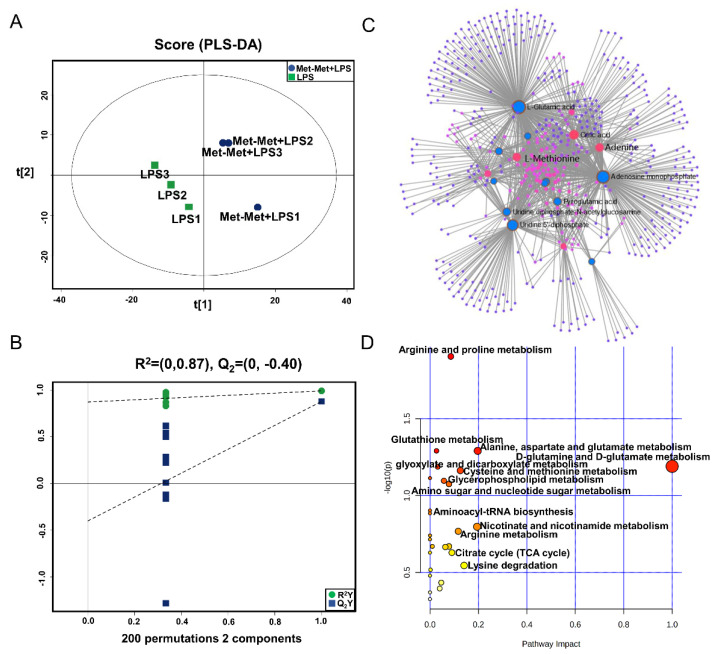
Met-Met altered the metabolic status of the inflammatory response. (**A**) The PLS-DA score plot of the Met-Met+LPS vs. LPS group. (**B**) Cross-validation plot of the PLS-DA model with 200 permutation tests in the Met-Met+LPS vs. LPS group. (**C**) Metabolite-metabolite interaction network of DMs in the comparative group of Met-Met+LPS vs. LPS. Node size represents the degree. Red and blue circles correspond to the increased and reduced DMs in the comparative group of Met-Met+LPS vs. LPS, respectively. (**D**) Pathway analysis of the DMs in the Met-Met+LPS and LPS groups. The *X*-axis and *Y*-axis indicate the pathway impact and the degree of pathway enrichment, respectively. A darker color indicates a higher degree of pathway enrichment, and a larger size indicates a higher pathway impact value. DMs: differential metabolites.

The PLS-DA (Figure 4A), OPLS-DA (Figure S1C), and 200 permutation test models (Figure 4B) of MAC-T in the comparative group of Met-Met+LPS and LPS showed that the cell metabolites of the Met-Met+LPS group were efficiently separated from those of the LPS group. Combined with VIP >1 and *p* < 0.1, 23 DMs were identified in the Met-Met+LPS vs. LPS group, of which 6 were upregulated and 17 were downregulated (Table S4). The most significant DMs were S-methyl-5’-thioadenosine (97-fold) and L-methionine (48-fold). Details of the DMs, including name, VIP, P-value, FC, M/Z, and Rt, are presented in Table S4. The correlation networks of DMs (Figure 4C) in the comparative group of Met-Met+LPS vs. LPS consisted of 540 nodes and 1198 edges. The most correlated DMs were L-methionine, L-glutamic acid, adenine, AMP, citrate, myristic acid, uridine 5’-diphosphate (UDP), and L-pyroglutamic acid. The KEGG pathway analysis (Figure 4D) showed that most metabolites were primarily involved in arginine and proline metabolism; glutathione metabolism; alanine, aspartate, and glutamate metabolism; D-glutamine and D-glutamate metabolism; cysteine and methionine metabolism, glyoxylate and dicarboxylate metabolism; aminoacyl-tRNA metabolism; and the citrate cycle (TCA cycle). 

### 3.5. Overlapping Metabolites and Pathways Regulated by Inflammation and Met-Met

**Figure 5:**
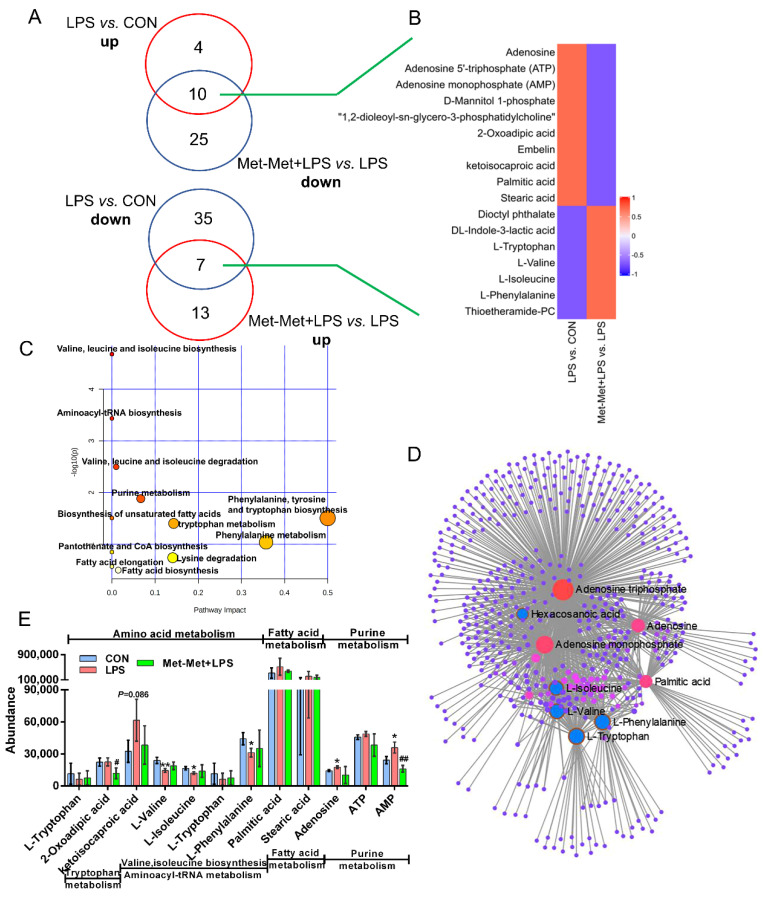
Overlapping metabolites and pathways regulated by inflammation and Met-Met. (**A**) Venn diagrams represent the DMs from two comparative groups of LPS vs. CON and Met-Met+LPS vs. LPS. (**B**) Heatmap of 17 overlapping metabolites. (**C**) Pathway analysis of 17 overlapping metabolites. The *X*-axis and *Y*-axis indicate the pathway impact and the degree of pathway enrichment, respectively. A darker color indicates a higher degree of pathway enrichment, and a larger size indicates a higher pathway impact value. (**D**) Metabolite-metabolite interaction network of overlapping metabolites. Node size represents the degree. The red circle represents the overlapping metabolites that were notably increased by LPS and further decreased by Met-Met. The blue circle corresponding to the overlapping metabolites was reduced in the LPS group and further upregulated by Met-Met. (**E**) The classification map of overlapping metabolites. * *p* < 0.05 and ** *p* < 0.01, represent the difference between LPS and CON group; ^#^ *p* < 0.05 and ^##^ *p* < 0.01 denote significance between the Met-Met+LPS and LPS groups; DMs: differential metabolites.

In total, 17 overlapping metabolites (VIP > 1) were identified. Among these metabolites, the overexpression of 10 overlapping metabolites was founded in LPS-induced inflammation, while their expression was downregulated by Met-Met (Figure 5A). In contrast, seven overlapping metabolites were identified with a decreased level in the LPS-induced inflammatory group, and their levels were reversed by Met-Met (Figure 5A,B). Details of the DMs, including name, VIP, P-value, FC, M/Z, and Rt, are presented in Table S5. In all, 522 nodes and 1143 edges were shown in the correlated network of all overlapping metabolites (Figure 5D). The correlation networks (Figure 5D) showed that the overlapping DMs were concentrated in adenosine triphosphate, AMP, adenine, palmitic acid, tryptophan, phenylalanine, valine, and isoleucine. Besides, KEGG pathway analysis (Figure 5C) showed that a majority of overlapping metabolites were dominantly enriched in valine, leucine, and isoleucine biosynthesis and degradation; aminoacyl-tRNA; purine metabolism; phenylalanine, tyrosine, and tryptophan metabolism; biosynthesis of unsaturated fatty acids; and elongation and biosynthesis of fatty acids. According to biochemical characteristics and biological processes, these DMs and metabolic pathways could be further divided into three categories: amino acid metabolism, purine metabolism, and fatty acid metabolism (Figure 5E).

## 4. Discussion

Bovine mastitis caused by lipopolysaccharide leads to severe economic loss for farmers [22]. In this research, an LPS-induced cellular inflammatory model was successfully established, confirmed by the increment of the cytokines *TNF-α* and *IL-8*, and the inflammatory mediators *AP-1* and *MCP-1*. Met-Met exhibited a suppressive effect on these proinflammatory cytokines under LPS exposure. Consistently, compared with the control group, Met-Met treatment alone significantly decreased the levels of *TNF-α, IL-8*, *AP-1,* and *MCP-1*. Although there is little literature about the suppressive effect of Met-Met on inflammation, our recent study found that Met-Met has a similarly inhibitive impact on the expression of *TNF-α* and *IL-8* [15]. Besides, our previous study demonstrated that free methionine could be produced by Met-Met through hydrolase aminopeptidase N [23]. Several investigations have also revealed that methionine exhibits a suppressive function on an LPS-induced increase of *TNF-α* and *IL-1β* in different types of bovine epithelial cells [24,25]. In this study, the content of L-methionine increased 57-fold and 48-fold in two comparative groups of Met-Met vs. CON and Met-Met+LPS vs. LPS, respectively. Therefore, Met-Met might ameliorate MAC-T inflammation via hydrolysate methionine. In addition, the inflammatory response was a dynamic balance process: the decreased *IL-8* might impair neutrophil migration, leading to greater *E. coli* invasion; therefore, the animal model needed to be further conducted to determine the optimal concentration of Met-Met. 

Amino acids play a fundamental role in the inflammatory response. The synthesis of antigen-presenting molecules, immunoglobulins, and cytokines in the innate and acquired immune systems requires amino acids as materials, and individual amino acids participate directly or indirectly in the immune response through their intermediates, such as histamine, glutathione (GSH), nitric oxide (NO), and superoxide [26,27,28]. Methionine and cysteine are crucial to protein synthesis in the immune system [29]. Methionine, which is a substrate for the synthesis of phosphatidylcholine and acetylcholine, plays an important role in the inflammatory response [30]. In our study, the content of methionine in the two groups of Met-Met vs. CON and Met-Met+LPS vs. LPS significantly increased, suggesting that Met-Met might be hydrolyzed into a single methionine. Moreover, methionine can be converted into cysteine through the trans-sulfide pathway, and cysteine is an important antioxidant precursor of GSH and H_2_S in cells [31]. GSH can also scavenge free radicals and other reactive oxygen species (such as hydroxyl radicals and hydrogen peroxide) and combine with a variety of exogenous substances for detoxification [32]. There is additional evidence that GSH could respond to immune challenges by regulating the NFκB signaling pathway [33], and the lack of cysteine or intracellular GSH can lead to a decrease in the number of CD4 cells and reduce cytotoxic T cell activity [34]. The differential metabolites in two comparative groups of Met-Met vs. CON and Met-Met+LPS vs. LPS were commonly enriched in the methionine and cysteine metabolism and glutathione metabolism pathways, suggesting that Met-Met might augment the inflammatory response through its intermediates methionine, cysteine, and GSH. Further experiments of the detection of cysteine concentration, GSH synthase activity, reactive oxygen species, and other oxidative defense proteins need to be performed. Tryptophan generates anthraquinone acid through the indoleamine 2,3-dioxygenase (IDO) pathway when cells are stimulated by lipopolysaccharide or cytokines [35]. In our study, the level of tryptophan was reduced in the group of LPS vs. CON and Met-Met+LPS vs. LPS, indicating that LPS might cause the catabolism of tryptophan. However, this trend was reversed by the treatment with Met-Met, suggesting that Met-Met might improve the inflammatory response via promoting the anabolism of tryptophan. Interestingly, a previous study showed that the tryptophan mediators serotonin, melatonin, and N-acetyl serotonin could inhibit the production of superoxide, scavenge free radicals, and attenuate *TNF-α* to enhance host immunity [36]. An important function of phenylalanine is to upregulate the activity of GTP cyclohydrolase I and to activate macrophages and leukocytes to produce NO, which exhibits an anti-inflammatory effect by inhibiting the tricarboxylic acid cycle, electron transfer, and DNA synthesis [37]. In the LPS vs. CON and Met-Met+LPS vs. LPS group, LPS treatment induced a decrease of phenylalanine, whereas the content of phenylalanine was adjusted by Met-Met treatment, implying that Met-Met might contribute to an inflammatory response by promoting the synthesis of phenylalanine.

Histidine creates histamine through decarboxylase in the mammary gland [38]. Histamine is the dominant mediator of the inflammatory response and can regulate a variety of physiological and immune functions by activating histamine receptors located on target cells [39]. Moreover, histamine could contribute to regulating platelet aggregation and regulating Th2 cell activity by reducing *IL-12* and increasing *IL-10* production [40]. Our results demonstrated that LPS significantly reduced the production of histidine in the LPS vs. CON group, speculating that bovine mastitis caused by Gram-negative bacteria was potentially accompanied by decreased histidine.

Purine metabolism is associated with amino acid metabolism through the purine nucleotide cycle and plays an important role in energy supply, metabolic regulation, and coenzyme production [41]. Purine metabolism includes the de novo purine synthesis pathway, salvage pathway, and degradation pathway [42]. Among them, the salvage pathway uses hypoxanthine, inosine, and adenine as substrates to generate purine nucleotides, while the degradation pathway further oxidizes inosine and hypoxanthine to xanthine and uric acid [42]. In our study, adenine and AMP were upregulated in the LPS vs. CON group, whereas inosine 5′-monophosphate (IMP), hypoxanthine, and inosine were downregulated. These results indicated that LPS disturbed the process of purine metabolism in MAC-T and tended to synthesize adenine and its nucleotides. Uric acid, a final product of purine metabolism, can neutralize pro-oxidant molecules, such as hydroxyl radicals, hydrogen peroxide, and peroxynitrite, in cells [43]. In the current research, adenine was augmented, whereas adenine nucleotides were decreased in the Met-Met vs. CON group, implying that Met-Met was more inclined to degrade purines, and its metabolite uric acid might exert an anti-inflammatory effect by scavenging free radicals.

Fatty acids can regulate the immune metabolism of animals and cells by affecting the intensity and duration of inflammation. Palmitic acid is a saturated fatty acid that can activate the NFκB pathway through toll-like receptors and generate reactive oxygen species to cause endoplasmic reticulum stress [44]. Additionally, Joshi-Barve et al. [45] found that palmitic acid could induce neutrophils to produce the cytokine *IL-8*. In our study, LPS induced a significant upregulation of palmitic acid, whereas this trend was adjusted by treatment with Met-Met, suggesting that Met-Met might relieve inflammation via the palmitic acid metabolism pathway. Similarly, stearic acid can aggravate inflammation by inducing superoxide to activate NLRP3 inflammasomes [46]. In our study, LPS induced a significant upregulation of stearic acid; however, treatment with Met-Met reduced the content of stearic acid in the LPS vs. CON and Met-Met+LPS vs. LPS group, suggesting that Met-Met could ameliorate MAC-T inflammation via metabolic alteration of stearic acid.

## 5. Conclusions

**Figure 6:**
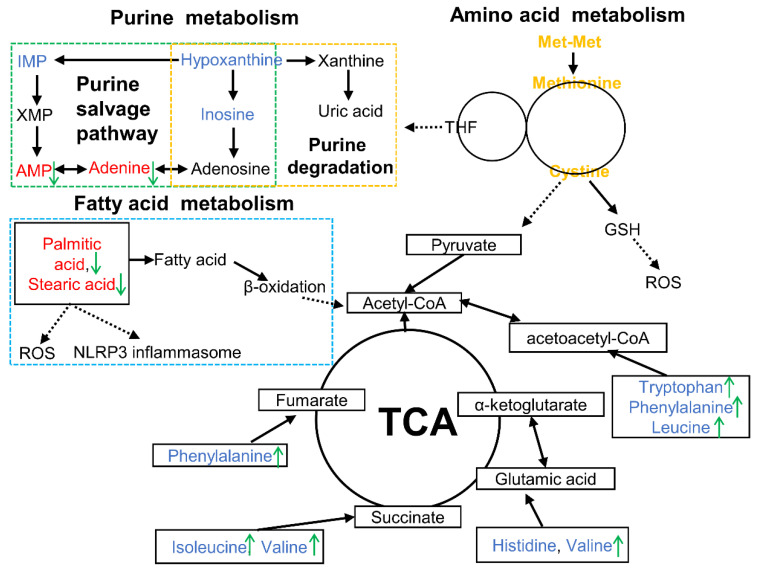
Overview of metabolic alterations related to Met-Met alleviating bovine mammary epithelial cell inflammation. Note: The red font represents increased DMs, while the blue font means the decreased DMs in the LPS vs. CON comparison. The green arrows indicate the changed DMs in the Met-Met+LPS vs. LPS group. AMP, adenosine monophosphate; DMs: differential metabolites; GSH, glutathione; IMP, inosine 5′-monophosphate; ROS, reactive oxygen species; TCA, tricarboxylic acid cycle; THF, tetrahydrofolate; XMP, xanthosine monophosphate.

This study demonstrated that Met-Met alleviated MAC-T inflammation via hydrolysate methionine. Methionine and cysteine metabolism, purine metabolism, and fatty acid metabolism could be responsible for the inflammatory response of Met-Met (Figure 6). This research provides the possibility to develop peptide additives to improve animal immunity.

## Supplementary Materials

The following are available online at https://www.mdpi.com/2076-2615/11/3/833/s1, Table S1: Primers used in mRNA abundance analysis, Table S2: DMs identified in MAC-T between the Met-Met and CON groups, Table S3: DMs identified in MAC-T between the LPS and CON groups, Table S4: DMs identified in MAC-T between the Met-Met+LPS and LPS groups, Table S5: The overlapped DMs identified in the LPS vs. CON and Met-Met+LPS vs. LPS groups, Figure S1: Overview of OPLS-DA and permutation tests in different comparative groups.
