# Supplementary material for: Metabolic Profile Reveals the Immunosuppressive Mechanisms of Methionyl-Methionine in Lipopolysaccharide-Induced Inflammation in Bovine Mammary Epithelial Cell
Source: Animals (Basel). 2021 Mar 16;11(3):833. doi: 10.3390/ani11030833 (PMC8000761; doi:10.3390/ani11030833)
Supplement: Supplementary file 1 [file animals-11-00833-s001.pdf]

Supplementary Materials

Metabolic Profile Reveals the Immunosuppressive Mechanisms of Methionyl-Methionine in LPS-Induced Bovine Mammary Epithelial Cell Inflammation by Lan et al.

Table S1. Primers used in mRNA abundance analysis

Gene	Primers (5'-3')	bp	Gene No.
TNF-α-F	CCACGTTGTAGCCGACATC	156	NM_173966.3
TNF-α-R	CCCTGAAGAGGACCTGTGAG		
IL-8-F	ATGACTTCCAAGCTGGCTGTTG	149	NM_173925.2
IL-8-R	TTGATAAATTTGGGGTGGAAAG		
AP-1-F	TCGTTCCTCCAGTCTGAGAGC	230	NM_001077827.1
AP-1-R	GTCGGCGTGGTGGTGATATG		
MCP-1-F	GACCTCCGCTGTCTTTCCAG	188	NM_ 174006.2
MCP-1-R	CTCGACGGCAACCATACACT		
RPS9-F	ATGAGGGCAAGATGAAGCTG	172	DT860044
RPS9-R	ATGAAGGACGGGATGTTCAC		

Supplementary Materials

Table S2. DMs identified in MAC-T between the Met-Met and CON groups

Name	VIP	FC	P-value	M/Z	Rt (s)
Uridine 5'-monophosphate (UMP)	1.043	0.464	0.00660	325.043	438.373
Uridine 5'-diphosphate (UDP)	1.259	0.591	0.05776	405.009	474.997
Nicotinamide	1.434	0.581	0.08296	123.055	85.3670
Adenosine monophosphate (AMP)	3.780	0.352	0.04151	348.070	427.012
S-Methyl-5'-thioadenosine	5.640	27.056	0.03266	298.097	98.7670
Adenine	2.699	3.532	0.08405	136.061	98.8200
Phenylacetic acid	1.441	10.842	0.00004	119.049	35.2730
L-Methionine	4.158	57.006	0.00083	150.058	280.601

Note: DMs：Differential metabolites; VIP: Variable important in projection; FC: Fold change; M/Z: Mass-to-charge ratio; Rt (s): Retention time.

Supplementary Materials

Table S3. DMs identified in MAC-T between the LPS and CON groups

Name	VIP	FC	P-value	M/Z	Rt (s)
Adenosine monophosphate (AMP)	1.519	1.487	0.07729	346.055	426.987
Adenine	1.001	1.236	0.07776	136.061	277.476
L-Histidine	1.010	0.761	0.03316	156.076	382.385
D-Fructose 1,6-bisphosphate	2.586	0.183	0.06357	322.993	499.089
L-Palmitoylcarnitine	1.509	0.283	0.04441	400.341	49.2520
Pyridoxine	5.167	0.554	0.00235	170.081	105.628
Inosine	1.527	0.067	0.00016	269.087	213.391
Hypoxanthine	6.333	0.063	0.00030	137.046	213.418
L-Leucine	1.664	0.736	0.05414	132.102	259.162
L-Isoleucine	1.502	0.733	0.04544	132.102	270.655
1-Oleoyl-sn-glycero-phosphocholine	1.055	0.612	0.02314	522.354	151.434
Phosphorylcholine	7.358	0.831	0.06081	184.074	472.931
Inosine 5'-monophosphate (IMP)	1.031	0.503	0.03094	347.039	444.065
1-Aminocyclopropanecarboxylic acid	2.735	0.629	0.06859	84.0450	395.116

Note: DMs: Differential metabolites; VIP: Variable important in projection; FC: Fold change; M/Z: Mass-to-charge ratio; Rt (s): Retention time.

Supplementary Materials

Table S4. DMs identified in MAC-T between the Met-Met+LPS and LPS groups

Name	VIP	FC	P-value	M/Z	Rt (s)
Dioleoyl glycero-phosphatidylcholine	2.178	0.622	0.03288	786.599	47.3820
Dehydroabietic acid	1.567	0.337	0.00071	299.201	41.0760
Adenosine monophosphate (AMP)	3.133	0.448	0.00465	348.070	427.012
L-Glutamate	2.688	0.657	0.09015	148.061	395.116
Glycerophosphocholine	1.596	0.485	0.02693	258.110	381.624
Phosphorylcholine	10.99	0.665	0.07060	184.074	472.931
Nicotinamide	3.189	0.510	0.04967	123.055	49.9930
D-Proline	1.104	0.636	0.09664	116.070	308.240
Phosphocreatine	3.086	0.563	0.05336	212.043	441.358
Uridine 5'-diphosphate (UDP)	1.117	0.633	0.07721	405.009	444.491
Uridine diphosphate glucose	1.453	0.599	0.08054	565.047	443.419
UDP-N-acetylglucosamine	1.438	0.627	0.09752	608.088	431.362
L-Glutamine	3.506	0.603	0.05761	145.062	370.477
Dihydrothymine	1.479	0.626	0.06389	127.051	370.276
1-Aminocyclopropanecarboxylic acid	3.522	0.658	0.06649	84.0450	367.393
L-Pyroglutamic acid	4.594	0.655	0.06676	147.077	367.393
2-Oxoadipic acid	1.474	0.524	0.04250	141.017	364.114
Myristic acid	1.078	2.874	0.06070	227.201	100.369
L-Methionine	3.665	48.240	0.01784	150.058	280.601
Citrate	1.704	2.174	0.03336	191.019	483.621
S-Methyl-5'-thioadenosine	6.105	97.769	0.00086	298.097	98.7670
Adenine	6.449	3.829	0.02997	136.062	159.466
Betaine	2.076	1.187	0.06715	118.086	441.872

Note: DMs：Differential metabolites; VIP: Variable important in projection; FC: Fold change; M/Z: Mass-to-charge ratio; Rt (s): Retention time.

Supplementary Materials

Table S5. The overlapped DMs identified in the LPS vs. CON and Met-Met+LPS vs. LPS groups

Name	M/Z	LPS vs. CON		Met-Met+LPS vs. LPS	
		VIP	FC	VIP	FC
Adenosine	268.104	1.001	1.236	1.681	0.584
Adenosine 5'-triphosphate (ATP)	508.003	2.331	1.066	1.238	0.787
Adenosine monophosphate (AMP)	346.055	1.519	1.487	3.133	0.448
D-Mannitol 1-phosphate	321.173	3.660	34.297	2.120	0.499
Phosphatidylcholine	808.584	2.539	1.186	2.178	0.622
2-Oxoadipic acid	141.017	4.103	1.007	1.474	0.524
Embelin	293.179	10.713	46.866	6.913	0.619
Ketoisocaproic acid	129.055	2.547	1.897	1.732	0.622
Palmitic acid	255.234	7.170	1.623	4.154	0.733
Stearic acid	283.264	5.530	2.181	3.505	0.821
Dioctyl phthalate	391.284	2.580	0.536	1.132	1.147
DL-Indole-3-lactic acid	188.071	1.656	0.685	1.268	1.070
L-Tryptophan	203.082	1.086	0.561	1.011	1.176
L-Valine	116.072	1.402	0.605	2.197	1.301
L-Isoleucine	132.102	1.502	0.733	1.432	1.150
L-Phenylalanine	166.086	2.529	0.704	2.222	1.130
Thioetheramide-PC	780.556	1.550	0.836	2.614	1.717

Note: DMs：Differential metabolites; VIP: Variable important in projection; FC: Fold change; M/Z: Mass-to-charge ratio; Rt (s): Retention time.

Supplementary Materials

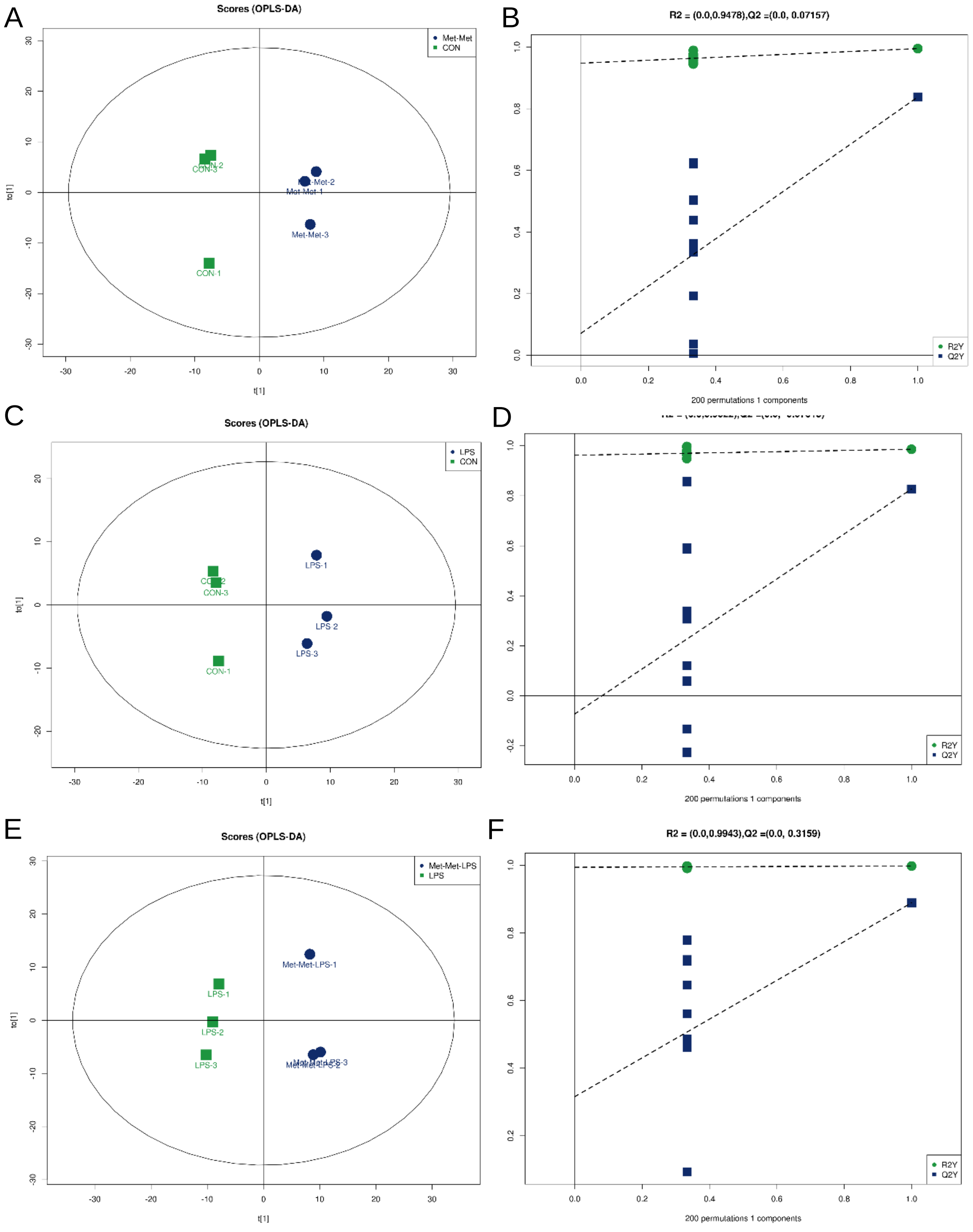

Figure S1. Overview of OPLS-DA and permutation tests in different comparative groups. A. The OPLS-DA score scatter plot of the Met-Met vs. CON group; B. Cross-validation plot of OPLS-DA model with 200 permutation tests in the Met-Met vs. CON group. C. The OPLS-DA score scatter plot of the LPS vs. CON group; D. Cross-validation plot of OPLS-DA model with 200 permutation tests in the LPS vs. CON group; E. The OPLS-DA score scatter plot of the Met-Met+LPS vs. LPS group. F. Cross-validation plot of OPLS-DA model with 200 permutation tests in the Met-Met+LPS vs. LPS group.
